# Response to “Letter to the editor regarding the Paper ‘S2k-Guideline hand antisepsis and hand hygiene’”

**DOI:** 10.3205/dgkh000667

**Published:** 2026-07-24

**Authors:** Axel Kramer, Andreas Enz

**Affiliations:** 1 Institute of Hygiene and Environmental Medicine, University Medicine Greifswald, Germany; 2 Helios Hospital Schwerin, Academic Teaching Hospital MSH Medical School Hamburg, Germany

## Dear author,

We appreciate the comment regarding the conceptual distinction between standard double gloving strategies and the use of indicator glove systems [1]. As this represents an important methodological aspect in guideline development as well as in surgical practice, the authors would like to clarify the underlying intention of the original comment and briefly summarize the available evidence.

The original comment by Mr. Schäfer was not intended to question the validity of the evidence supporting indicator glove systems. Rather, it addressed whether the outcome parameter used in Article 47 of the S2k guideline adequately reflects the distinct mechanism of benefit associated with indicator systems.

The inner glove perforation rate primarily reflects barrier integrity and is therefore an appropriate endpoint for evaluating standard double-gloving strategies. Indicator glove systems, in contrast, provide their main benefit through early intraoperative perforation recognition and the resulting timely glove change. From a guideline methodology perspective, distinguishing between barrier-based protection and detection-based risk mitigation is important, as these mechanisms may require different endpoints and may lead to more differentiated recommendations.

The following measures may reduce the risk of surgical glove breach:

- Proper glove fit [2],
- double gloving [3], [4],
- use of indicator gloves [5], [6], [7], and
- timely glove change [8].

When single gloves are used, glove change after visible perforation or at predefined time intervals may help maintain the surgical barrier. While no generally accepted protocol had previously been available, the recently published international consensus now provides specific indication- and situation-dependent recommendations for glove change, suggesting replacement after 60–120 minutes at the latest [7].

Three systematic reviews have shown substantially improved perforation detection with indicator glove systems [5], [6], [7]. The most recent review included 32 studies were included, among them multiple high-quality level I trials [6]. Across studies, indicator systems provided a two- to six-fold higher rate of perforation detection compared with standard double gloving using two gloves of the same colour [6]. This supports the conclusion that indicator systems offer a distinct safety benefit through timely perforation recognition and glove change, rather than through barrier reinforcement alone. Evidence for downstream patient-centred outcomes such as SSI remains limited, whereas the evidence for improved perforation detection and maintenance of barrier function is substantially stronger. In summary, indicator glove systems provide a distinct safety benefit by enabling timely perforation recognition and glove change, rather than by strengthening the barrier itself. We therefore agree that indicator glove systems should not simply be evaluated by the same outcome parameters used for standard double gloving. Their principal benefit lies in perforation detection and timely glove change, and this distinction should be reflected in guideline interpretation and recommendation development.

In any case glove change before implantation procedures is necessary, particularly in settings where contamination of initially sterile gloves has been demonstrated [9], [10]. In such situations, indicator systems may be helpful because they support rapid recognition of glove perforation and timely glove replacement. We therefore agree that indicator glove systems should not simply be evaluated by the same outcome parameters used for standard double gloving. Their principal benefit lies in perforation detection and timely glove change, and this distinction should be reflected in guideline interpretation and recommendation development. The reason is that the gloves are contaminated by the surgical field because preoperative skin antisepsis does not reach the deep skin flora, since alcohol-based solutions do not penetrate the hair follicles [11]. When double gloves are worn with an indicator system and the system does not show any perforations, the advantage is that only the outer glove needs to be replaced. Otherwise, both gloves must be replaced. In this case, aseptic technique can be maintained with only a brief interruption of the surgical procedure.

## Notes

### Competing interests

The authors declare that they have no competing interests.

### Funding

None.

### Authors’ ORCIDs

- Kramer A: https://orcid.org/0000-0003-4193-2149
- Enz A: https://orcid.org/0000-0002-9258-487X
